# Conjunctival Goblet Cell Responses to TLR5 Engagement Promote Activation of Local Antigen-Presenting Cells

**DOI:** 10.3389/fimmu.2021.716939

**Published:** 2021-08-09

**Authors:** Abiramy Logeswaran, Laura Contreras-Ruiz, Sharmila Masli

**Affiliations:** Department of Ophthalmology, Boston University School of Medicine, Boston, MA, United States

**Keywords:** conjunctiva, ocular mucosa, goblet cells, homeostasis, dendritic cells

## Abstract

Conjunctival epithelium forms a barrier between the ocular surface microbial flora and the ocular mucosa. In addition to secreting gel-forming mucins, goblet cells, located in the conjunctival epithelium, help maintain local immune homeostasis by secreting active TGFβ2 and promoting tolerogenic phenotype of dendritic cells in the vicinity. Although dendritic cell subsets, characteristic of mucosal tissues, are found in the conjunctiva, previous studies provided limited information about their location within the tissue. In this study, we examine immunostained conjunctiva explants to determine the location of CD11c-positive dendritic cells in the context of MUC5AC-positive goblet cells. Considering that conjunctival goblet cells are responsive to signaling induced by pathogen recognition receptors, we also assess if their responses to microbial product, flagellin, can contribute to the disruption of ocular mucosal homeostasis that promotes activation of dendritic cells and results in chronic ocular surface inflammation. We find that dendritic cells in the conjunctiva with an increased microbial colonization are located adjacent to goblet cells. While their cell bodies in the stromal layer are immediately below the epithelial layer, several extensions of dendritic cells are projected across the epithelium towards the ocular surface. Such trans-epithelial dendrites are not detectable in healthy ocular mucosa. In response to topically applied flagellin, increased proportion of CD11c-positive cells in the conjunctiva strongly express MHC class II relative to the untreated conjunctiva. This change is accompanied by reduced immunoreactivity to TGFβ-activating Thrombospondin-1 in the conjunctival epithelium. These findings are supported by *in vitro* observations in primary cultures of goblet cells that respond to the TLR5 stimulation with an increased expression of IL-6 and reduced level of active TGFβ. The observed changes in the conjunctiva after flagellin application correspond with the development of clinical signs of chronic ocular mucosal inflammation including corneal epitheliopathy. Collectively, these findings demonstrate the ability of ocular mucosal dendritic cells to extend trans-epithelial dendrites in response to increased microbial colonization at the ocular surface. Moreover, this study provides key insight into how goblet cell responses to microbial stimuli may contribute to the disruption of ocular mucosal homeostasis and chronic ocular mucosal inflammation.

## Introduction

Ocular mucosa is persistently exposed to environmental factors including microbes. The epithelial layer of the ocular mucosa is composed of stratified columnar epithelial cells that are interspersed with goblet cells (GC) capable of secreting gel-forming mucins to form a physical barrier between the environment and host cells. At steady state, similar to other mucosal surfaces, it is critical that the ocular mucosal tissue environment supports immunologic tolerance to harmless microbes and protective immunity against pathogens to facilitate their rapid clearance. In addition to secreting mucins, conjunctival GC are found capable of contributing to maintaining a homeostatic balance between tolerogenic and protective immune responses. Several studies support such function of conjunctival GC. These include observations of GC loss in chronic ocular surface inflammatory conditions, their ability to secrete immunomodulatory factors, and loss of immunologic tolerance in mice deficient in GC (1–4). These reports and evidence from other mucosal surfaces suggest GC to be multi-faceted players in mucosal immunity (5).

In colonic mucosa, microbial sensing by GC is reported to limit the exposure of the colonic mucosal immune system to harmful antigens (6). Conjunctival GC are also known to be responsive to microbial products. Studies have reported their ability to secrete pro-inflammatory IL-1β in response to stimulation by toxigenic *S. aureus* (7) and anti-inflammatory TGFβ2 in response to LPS-mediated stimulation (8). Although these studies implicate their ability to modulate the tissue environment, it is not known if their responses can contribute to chronic ocular surface inflammation.

Previously, we have reported that conjunctival GC predominantly express TGFβ2 isoform and mediate its activation by their endogenously expressed thrombospondin-1 (TSP-1) (8). Consistently, TSP-1 deficiency in mice results in the spontaneous development of chronic ocular surface inflammation as seen in patients with Sjögren’s syndrome (9). In these mice as well as in Sjögren’s syndrome patients, increased microbial colonization is detected at the ocular surface (10, 11). Together, these studies point to TSP-1-mediated mechanisms as potential contributors to ocular mucosal homeostasis. In this study, we examine if GC responses to microbial products can alter the ocular mucosal tissue environment to disruption of homeostasis and whether such disruption can lead to the development of chronic ocular surface inflammation.

Under steady-state conditions, professional antigen-presenting cells like dendritic cells (DC) located in tissues are maintained in an immature tolerogenic phenotype characterized by low expression of MHC class II and co-stimulatory molecules. These cells function as sentinels capable of transporting captured antigens to draining lymph nodes to initiate an antigen-specific adaptive immune response. While in our previous studies we demonstrated the ability of GC-derived TGFβ2 to maintain immature DC phenotype *in vitro* (8), in this study, we examine if GC–microbial interaction-driven changes in ocular mucosal homeostasis alter DC phenotype *in vivo* that supports the development of chronic ocular surface inflammation.

Our results demonstrate that TLR5 engagement on GC downregulates their expression of TSP-1 both *in vitro* and *in vivo*. Resultant decline in GC-derived active TGFβ along with increased expression of IL-6 contribute to a disruption of ocular mucosal homeostasis. Such altered tissue environment supports the activated phenotype of DC in the conjunctiva and subsequent development of chronic ocular surface inflammation characterized by corneal epitheliopathy and tissue infiltrates.

## Materials and Methods

### Mice

Wild-type (C57BL/6) mice were purchased from Charles River Laboratories (Wilmington, MA). TSP-1^−/−^ mice were purchased from Jackson Laboratories (Bar Harbor, ME). They were then bred in-house at a pathogen-free facility at Boston University School of Medicine, Boston, MA. The experimental protocols strictly followed the ARVO Statement for the Use of Animals in Ophthalmic and Vision Research and were in accordance with the institution’s guidelines.

### Flagellin Treatment of Mice

Wild-type mice were treated topically with 10 ng flagellin (InvivoGen, San Diego, CA) bilaterally (5 µl/eye) for 7 days. Corneal barrier integrity was monitored for 4 weeks by fluorescein staining as described previously (12). Briefly, 1% sodium fluorescein (Sigma-Aldrich, St. Louis, MO, USA) was applied to each eye. Three minutes later, eyes were flushed with PBS to remove excess fluorescein, and corneal staining was evaluated using the slit-lamp microscope (Haag-Streit, USA) with cobalt blue light. Punctate staining was scored according to the standardized National Eye Institute grading system of 0–3 for each of the five areas of the cornea (13).

### Immunostaining and Microscopy

To prepare frozen sections of conjunctiva, eyes were enucleated with the lids intact, fixed in 4% formaldehyde in phosphate buffered saline (PBS) for 48 h, followed by cryoprotection in 10%–30% sucrose for 72 h before embedding in OCT. Tissue sections 7 µm in thickness were cut using a microtome and placed on slides to be stored at – 20°C until ready to use. Conjunctival explants were fixed in 4% formaldehyde before staining. Tissue sections and explants were blocked by PBS that contained normal goat, hamster, or donkey serum, 0.3%–1% Triton X-100 at RT for 1 h, and incubated overnight at RT with primary specific antibodies for CD11c (Biolegend, Dedham, MA), MUC5AC (Abcam, Cambridge, MA), MHC class II (BD Biosciences, San Jose, CA), or TSP-1 (Santa Cruz Biotech, Santa Cruz, CA). After a PBS rinse, fluorescence-conjugated secondary antibodies (Invitrogen, Thermo Fisher Scientific, Waltham, MA) were applied for 1 h in RT. Cell nuclei were counterstained with DAPI dye. Fluorescent staining in tissue sections was visualized using the FSX100 Olympus fluorescence microscope. Staining in conjunctival explants was visualized using a Zeiss LSM 700 confocal microscope equipped with a 63× oil objective. Images (z-stacks) were captured using the Zeiss ZEN software. Microscopic images were analyzed using NIH ImageJ software (14).

### Primary Cultures of Conjunctival Goblet Cells

Goblet cells were obtained from mouse conjunctival pieces and grown in an organ culture as described previously (15). Briefly, conjunctival tissues were removed from 4- to 12-week-old male mice and kept in Hank’s based salt solution (Lonza, Walkersville, MD). Small pieces of conjunctival tissue were anchored onto a scratch at the bottom of a well in a 24-well plate. Culture medium (RPMI-1640) supplemented with 10% heat-inactivated fetal bovine serum, 10 mM HEPES, 100 µg/ml penicillin/streptomycin, 1 mM sodium pyruvate, and 1× nonessential amino acid mixture (Lonza, Walkersville, MD) was added to submerge the tissue pieces. These explants were fed every other day with RPMI-1640 medium and grown for 2 weeks at 37°C and 5% CO_2_ to reach 85% confluence. Explants were later discarded, and goblet cell phenotype was confirmed by positive immunostaining for cytokeratin-7 and MUC5AC and negative staining for cytokeratin-4 (15). Goblet cells were cultured with heat-killed pathogenic *Pseudomonas aeruginosa* strain PA14 (kindly provided by Dr. Mihaela Gadjeva, Brigham and Women’s Hospital, Harvard Medical School, Boston, MA) at a MOI of 60 or flagellin-PA (InvivoGen, San Diego, CA) at different concentrations (0–10 µg/ml) for a period of 24 h. No significant effect on cell viability was detected as determined using CCK-8 kit (Dojindo Molecular Technologies, Inc., Rockville, MD). Culture supernatants were collected to determine cytokine content and cells were used to harvest RNA.

### Flow Cytometry

Cultured goblet cells were stained with Fixable viability dye (eBioscience, San Diego, CA) and cells were immunostained with Alexa-647-conjugated anti-TLR5 antibody (Clone ACT5, Biolegend, Dedham, MA). Specificity of TLR5 staining was validated by confirming negative staining in RAW 264.7 cell line consistent with their minimal TLR5 transcript levels and functional responses to TLR5 agonist (16, 17). Fluorescence-labeled cells were analyzed using BD LSRII Flow Cytometer (BD Bioscience, San Jose, CA) at Boston University Flow Cytometry Core Facility. Further analysis of the data was performed using FlowJo software (Tree Star, Inc., Ashland, OR).

### TGFβ Bioassay

Active TGFβ content of culture supernatants was determined using fibroblast cell line, MFB-11, derived from TGFβ knockout mice and stably transfected with SBE-SEAP reporter (18). Cells in complete DMEM were seeded into flat-bottomed 96-well plates (3 × 10^4^ per well) and incubated overnight at 37°C, 5% CO_2_. Cells were then washed twice with PBS and incubated with 50 µl of serum-free DMEM for 2 h before adding culture supernatants. Standard curve was set up using recombinant TGFβ2 (R&D Systems, Minneapolis, MN). After 24 h incubation, culture supernatants from each well were tested for SEAP activity using Great EscApe SEAP Reporter system 3 (Clontech, Mountain View, CA) according to the manufacturer’s instructions. Absorbance was measured using a Synergy H1 microplate reader (Biotek, Winooski, VT). Each sample was analyzed in triplicate.

### Real-Time PCR

Total RNA was isolated using TRIzol Reagent (Life Technologies, Carlsbad, CA) according to the manufacturer’s directions and reverse transcribed to generate cDNA using the Superscript VILO cDNA kit (Life Technologies, Carlsbad, CA). Real-time PCR was performed to determine relative quantitative expression levels of TSP-1, TGFβ2, and IL-6 on 7200 Real-Time System (Applied Biosystems, Carlsbad, CA) using SYBR Green PCR Master Mix (Life Technologies, Carlsbad, CA). Amplification reactions were set up in quadruplicate using specific primer sets with the thermal profile of 95°C for 3 min, 40 cycles of 95°C for 20 s, 55°C for 30 s, and 72°C for 40 s. A melting curve analysis was performed to verify the specificity of amplification reactions. Analysis of fluorescence signal generated at each cycle with system software generated threshold cycle values, and quantification of gene expression was determined relative to that of the reference gene Glyceraldehyde-3-phosphate dehydrogenase. Primer sequences used were TGFβ2 (F-5’-AGGCGAGATTTGCAGGTATTGA-3’ and R-5’-GTAGGAGGGCAACAACATTAGCAG-3’), TSP-1 (F-5’-AAG AGG ACC GGG CTC AAC TCT ACA and R-5’-CTC CGC GCT CTC CAT CTT ATC AC), IL-6 (F-5’-AGT CAA TTC CAG AAA CCG CTA TGA and R-5’-TAG GGA AGG CCG TGG TTG T), and GAPDH (F-5’-CGA GAA TGG GAA GCT TGT CA and R-5’-AGA CAC CAG TAG ACT CCA CGA CAT).

### Statistical Analysis

Differences between mean values were compared using Student’s *t*-test. A difference with *p* < 0.05 is considered statistically significant. The standard error of the mean is represented with error bars in figures. For corneal fluorescein staining scores, normal distribution of the pooled data for independent groups from a series of experiments was confirmed using D’Agostino and Pearson test supporting the assumption that data in this study are drawn from a normally distributed population. Homogeneity of variances among experimental and control groups are confirmed using *F*-test. Data analysis was performed using Excel (Microsoft Office) and GraphPad Prism software (GraphPad software Inc., San Diego, CA).

## Results

### Dendritic Cells in the Conjunctiva With Increased Microbial Frequency Are Located in Close Proximity of Goblet Cells and Extend Dendrites to Access Ocular Surface

**Figure 1:**
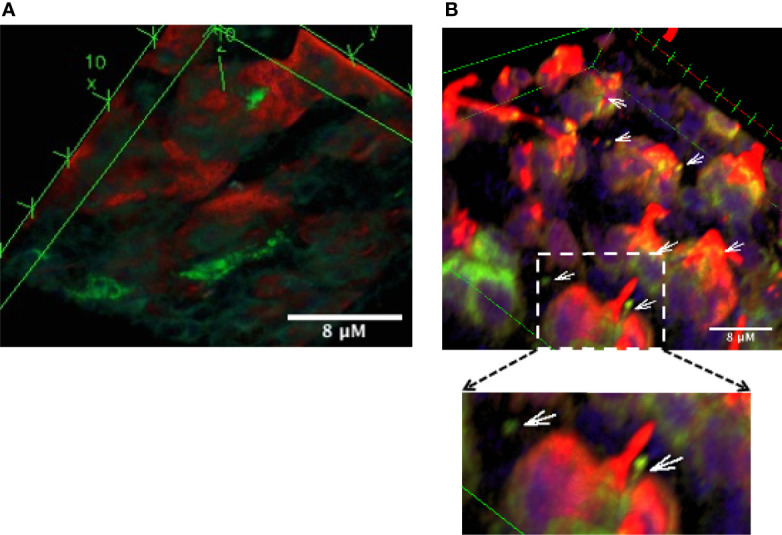
Dendritic cells are located in close proximity of conjunctival goblet cells and extend trans-epithelial dendrites towards the ocular surface. Confocal microscopy images of the conjunctival explant from TSP-1^−/−^ mice with increased microbial frequency at the ocular surface. Tissue was stained for MUC5AC (red) to label GCs and CD11c (green) to label DCs. Images show three-dimensional view of 63 z-stack images captured using laser-scanning confocal microscope. **(A)** Bottom view of the explant shows sub-epithelial location of some DCs (green), **(B)** In the top view, extensions of DCs (arrows) and their proximity of GCs (red) can be observed. Digital magnification of the boxed region in the top panel is shown to better illustrate DC extension with a globular tip protruding towards the ocular surface.

Similar to other mucosal surfaces, the presence of CD11c+ DC subsets have been reported in mouse ocular mucosa (19). These were identified by flow cytometry. In the intestinal mucosa, DC in the lamina propria extend trans-epithelial dendrites in response to epithelial cell TLR engagement (20). To evaluate the location and morphology of DC in the context of GC in the ocular mucosa, we evaluated conjunctiva explants from TSP-1^−/−^ mice, previously reported to harbor increased microbial frequency at the ocular surface (11), and from C57BL/6 mice as controls. A representative 3D reconstruction of confocal images of immunostained conjunctiva from TSP-1^−/−^ mice is shown in **Figure 1**. Positively stained cell bodies of DC were detected below the epithelial layer containing MUC5AC+ GC (**Figure 1A**). While several DC were located in close proximity of GC, many DC extensions were visible on the ocular surface side of the tissue (**Figure 1B**). Some of these extensions had globular structure at the tip similar to that described by others in trans-epithelial dendrites (TEDs) of DC subsets in intestinal mucosa (20–22). In C57BL/6 conjunctiva, very few positively stained DC were located in the subepithelial region without clearly detectable trans-epithelial projections (**Supplemental Figure 1**). The presence of TEDs in TSP-1^−/−^, but not C57BL/6, conjunctiva correlates with the known increased microbial colonization of the ocular surface in TSP-1^−/−^ mice. These results demonstrate that DC in ocular mucosa can be located in close proximity of GC and extend trans-epithelial dendrites towards the ocular surface presumably to sample microbes.

### Goblet Cells Express Functional TLR5, Which Mediates Reduced Secretion of Active TGFβ

**Figure 2:**
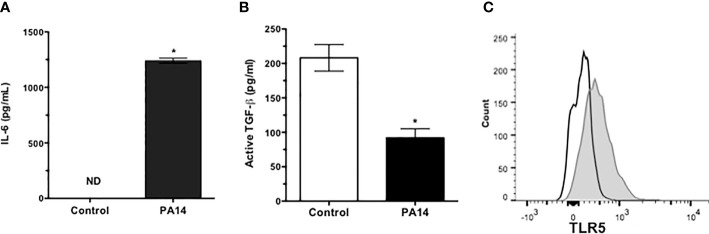
Goblet cells express functional TLR5 and respond to pathogenic strain of *P. aeruginosa* by reducing active TGFβ secretion. Levels of **(A)** IL-6 and **(B)** active TGF**β** in culture supernatants of GCs stimulated with heat-inactivated PA14 for 24 h. ND, Not Detected. Data are expressed as mean ± SEM, *n* = 4, *p < 0.05. **(C)** Flow cytometric detection of surface expression of TLR5 in primary cultures of GCs. Filled histogram shows positively stained GCs and empty histogram represents isotype staining control.

Microbial keratitis caused by *P. aeruginosa* is the most common vision-threatening infection among contact lens wearers (23) and a relatively common but potentially serious cause of conjunctivitis in hospitalized preterm infants with significant morbidity and in some cases death due to systemic complications (24). Potential complications of keratitis include chronic inflammation and corneal scarring. To determine if GC responses may contribute to these complications, we examined their responses to pathogenic strain of *P. aeruginosa* (PA14). We stimulated primary GC cultures, derived from C57BL/6 conjunctiva explants, with heat-inactivated PA14 and detected significantly increased levels of IL-6 in culture supernatants (**Figure 2A**). As GC respond to LPS (TLR4 ligand) by increasing their secretion of active TGFβ, we determined if PA14 induces a similar response in GC. As shown in **Figure 2B**, GC stimulated with PA14 produced significantly reduced levels of active TGFβ as detected in a bioassay described in Materials and Methods.

**Figure 3:**
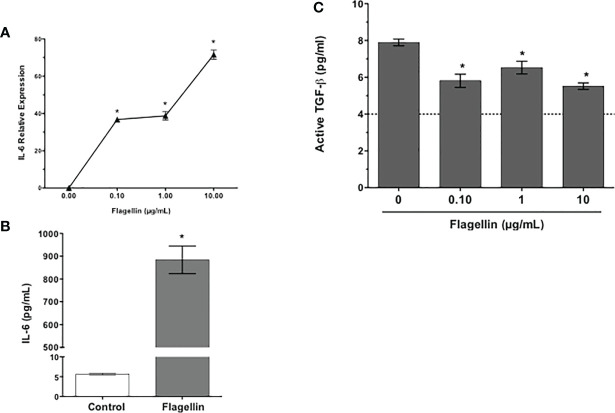
Flagellin, a TLR5 ligand, reduces GC secretion of active TGFβ. Cultures of GCs were stimulated with indicated concentrations of flagellin for 24 h. The expression of IL-6 message **(A)** was detected by real-time PCR and IL-6 protein levels were determined by ELISA **(B)** in culture supernatants of GCs stimulated with 1 µg/ml flagellin. Culture supernatants were also evaluated for their content of active TGFβ **(C)** in a bioassay. The dotted line represents assay limit of detection. Data are expressed as mean ± SEM, *n* = 4, *p < 0.05.

Conjunctival GC are known to respond to TLR2- and TLR4-mediated signals (7, 8). To determine their TLR5 expression, cells from primary GC cultures were stained with anti-TLR5 antibody and analyzed by flow cytometry. Consistent with reported expression of TLR5 in human conjunctival epithelial cells (25, 26), GC expressed TLR5 (**Figure 2C**). To determine if reduced TGFβ secretion induced by PA14 resulted from combined TLR4/5 signaling or if it can be attributed to TLR5-mediated signaling in GC, we stimulated these cells with different concentrations of TLR5 ligand, flagellin derived from *P. aeruginosa*. As shown in **Figure 3**, we detected dose-dependent increased expression of IL-6 message by real-time PCR (**Figure 3A**) in flagellin-stimulated GC that was accompanied by increased IL-6 protein detected in culture supernatants by ELISA (**Figure 3B**). Similarly, flagellin also reduced secretion of active TGFβ by GC in a dose-dependent manner (**Figure 3C**). Together, these results confirm the expression of functional TLR5 on conjunctival GC that, in contrast to TLR4-mediated signals, reduces GC-derived active TGFβ.

### Flagellin-Mediated Modulation of Goblet Cell TGFβ Secretion Contributes to the Development of Chronic Ocular Surface Inflammation

**Figure 4:**
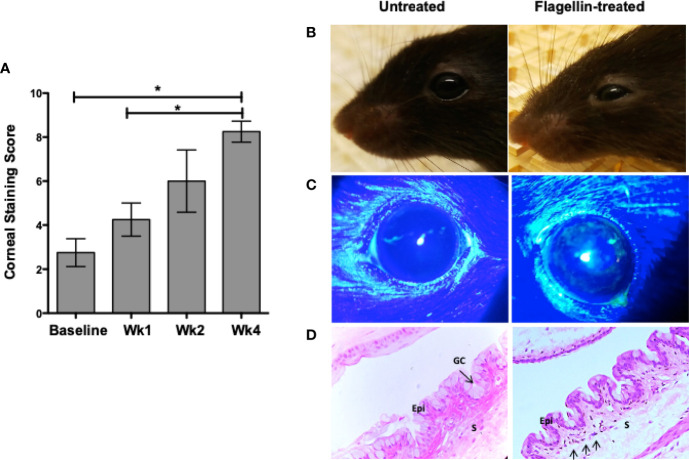
Topical application of flagellin results in chronic ocular surface inflammation. Flagellin (10 ng/day) was applied topically in C57BL/6 mice for 7 days. Development of ocular surface inflammation was monitored by assessing corneal barrier integrity, clinical signs, and conjunctiva histology. Corneas were evaluated for punctate epitheliopathy before and up to 4 weeks post-flagellin application using 1% sodium fluorescein application followed by slit lamp exam using cobalt blue light. Staining was scored according to standardized NEI grading system **(A)**. Representative photographs of untreated and flagellin-treated mice **(B)**; their corneal fluorescein staining **(C)**; and H&E-stained sections of conjunctiva at 4 weeks. **(D)** Epithelium (Epi), Stroma (S), and Goblet cells (GC) are marked, and arrows in the conjunctiva from flagellin-treated mouse point to inflammatory infiltrates. Data expressed as mean ± SEM, *n* = 4, *p < 0.05 wk4 *vs.* baseline.

The ability of GC to secrete TGFβ and modulate DC phenotype implicates a potential role of GC in maintenance of immune homeostasis (8). To determine if flagellin-mediated reduced secretion of active TGFβ and increased secretion of IL-6 by GC disrupt ocular mucosal homeostasis and lead to the development of chronic ocular surface inflammation, we topically administered flagellin (10 ng/mouse) in mice daily for 1 week. Chronic inflammation of the conjunctiva is associated with the development of corneal epitheliopathy resulting from a disrupted corneal barrier. Therefore, we assessed corneal barrier integrity by determining corneal fluorescein staining score before initiating flagellin application (baseline) and comparing it to scores determined up to 4 weeks post-flagellin application. As shown in **Figure 4A**, a gradual increase in corneal fluorescein score led to a significant increase by 4 weeks after completion of flagellin application as compared to the baseline score. Consistent with this result, clinical signs of conjunctival inflammation were noted in mice. These included edema and hair loss around the eye caused by excessive grooming (**Figure 4B**). **Figure 4C** shows representative corneal fluorescein staining in untreated and flagellin-treated mice. These clinical signs correlated with inflammatory infiltrates observed in histological evaluation of conjunctiva from flagellin-treated mice (**Figure 4D**). Also, GC density appeared to be lower in flagellin-treated conjunctiva relative to that seen in untreated tissue. Together, these findings indicate development of chronic ocular surface inflammation in mice treated with topical application of flagellin.

### Flagellin Stimulation of Goblet Cells Is Associated With Reduced Expression of TGFβ-Activator TSP-1 and Altered Ocular Mucosal Homeostasis

**Figure 5:**
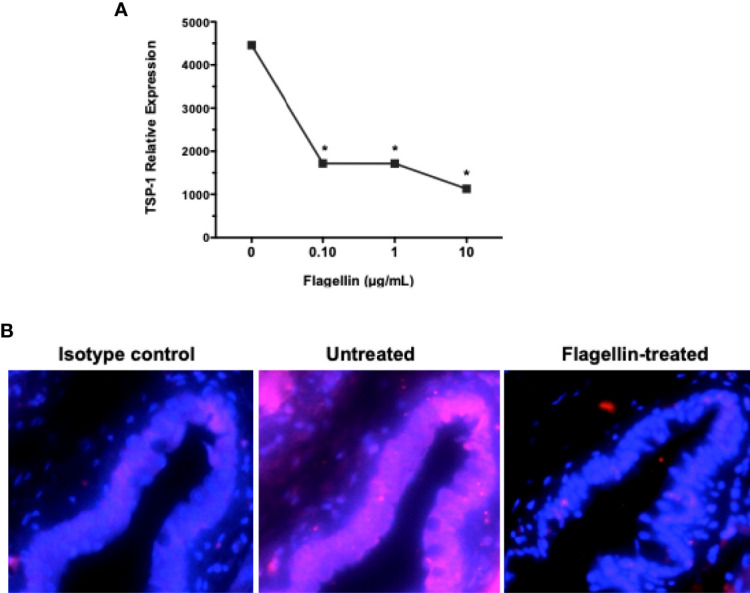
Flagellin stimulation is associated with reduced expression of TGFβ-activator TSP-1. **(A)** The expression of TSP-1 in GC cultures stimulated with indicated concentrations of flagellin was determined by real-time PCR. Data are expressed as mean ± SEM, *n* = 4, *p < 0.05. **(B)** Immunostaining of TSP-1 (magenta) in frozen sections of conjunctiva tissue harvested from C57BL/6 mice 4 weeks after topical application of flagellin (10 ng/day for 7 days). Nuclei were stained with DAPI (blue). Magnification ×20.

We have reported previously that GC depend on their endogenously expressed TSP-1 to activate their latent TGFβ (8). Moreover, TSP-1 deficiency in mice results in spontaneous development of chronic ocular surface inflammation (9). Therefore, we evaluated change in TSP-1 expression in primary cultures of GC exposed to different concentrations of flagellin. As shown in **Figure 5A**, significantly reduced levels of TSP-1 message were detected by real-time PCR in flagellin-treated GC. This *in vitro* finding was also confirmed *in vivo*, by assessing TSP-1 immunostaining in conjunctiva from flagellin-treated mice. **Figure 5B** shows reduced TSP-1 immunoreactivity in conjunctival epithelium after flagellin application.

**Figure 6:**
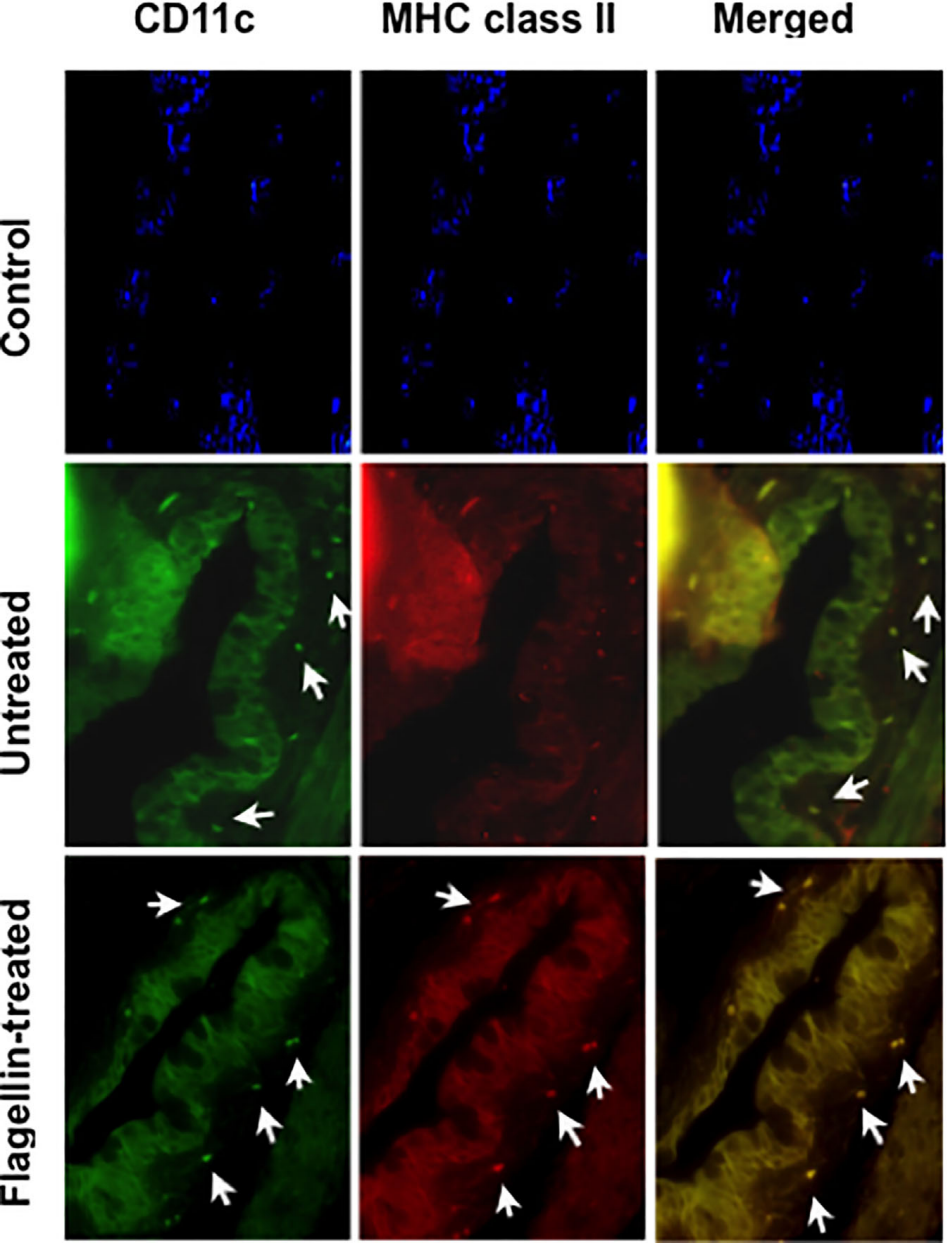
Topically applied flagellin increases proportion of MHC class II expressing DCs in the conjunctiva. Frozen sections of conjunctiva harvested from either untreated or flagellin-treated mice (10 ng/day for 7 days) were immunostained for dendritic cell marker CD11c and their activation marker MHC class II. Nuclei were stained with DAPI (blue). White arrows indicate positively stained cells. Magnification ×20.

Immature or tolerogenic state of DCs in tissues under steady-state homeostatic conditions is characterized by their low expression of MHC class II. To determine if flagellin-induced reduced TSP-1 and active TGFβ expression by GC alter ocular mucosal homeostasis, we examined MHC class II expression of CD11c+ DC in flagellin-treated conjunctiva. As seen in **Figure 6**, while CD11c+ DC are detectable in untreated conjunctiva, MHC class II expression of these cells was stronger in flagellin-treated conjunctiva. These results indicate loss of homeostasis in flagellin-exposed conjunctiva is consistent with reduced TSP-1 expression in this tissue.

## Discussion

As a mucosal surface persistently exposed to microbes, the steady-state homeostasis in the conjunctiva is central to ensuring immunologic tolerance induction while preventing excessive inflammatory damage to the tissue. Our results in this study demonstrate that DC in the conjunctiva can be located in close proximity of GC and can extend TEDs similar to those observed in other mucosal surfaces. Considering the location of DC, responses of GC to microbial stimuli have the potential to directly influence their functional phenotype. Notably, GC responses to the pathogenic strain of *P. aeruginosa* or TLR5-mediated signaling included altered balance in the expression of immunomodulatory TGFβ and pro-inflammatory IL-6. The decline in GC-derived active TGFβ induced by TLR5 stimulation correlated with their reduced expression of TGFβ-activator TSP-1. In mice, TLR5 agonist-induced altered tissue environment led to activated phenotype of DC indicated by their increased MHC class II expression and chronic ocular surface inflammation with corneal epitheliopathy.

Our results highlight the significance of GC–microbial interactions in maintaining homeostasis in the ocular mucosa. These findings implicate GC responses in altering tissue microenvironment in a way that promotes development of chronic inflammation. Although expression of TLR5 is reported in conjunctival epithelial cells (26), our data not only demonstrate its specific expression on GC but confirm flagellin-induced inflammatory IL-6 secretion in GC. In mice, macrophages and bone marrow-derived DC do not express TLR5, ruling out chronic inflammation resulting from systemic stimulation of DC (27). However, CD11c+ DC in intestinal mucosa express high levels of TLR5 in addition to intestinal epithelial cells. While normal conjunctiva in mice harbors CD11c+ DC, it is not known if these cells express TLR5 (19). Due to its size (>10 kDa), topically applied flagellin in our *in vivo* experiments is not expected to have crossed the epithelium through paracellular leak or GC-associated passages (GAPs), limiting the possibility of direct activation of conjunctival DC. Also, in normal conjunctiva, we did not observe TED formation (**Supplementary Figure 1**) in DC that could give them direct access to topically applied flagellin. However, TED formation induced by flagellin-stimulated GC or stratified epithelial cells cannot be ruled out. Regardless of cells targeted *in vivo* by flagellin, our experiments clearly support its ability to disrupt ocular mucosal homeostasis. Furthermore, the detection of corneal epitheliopathy over a prolonged period after stopping topical flagellin administration highlights the chronic nature of the ocular surface inflammation.

Conjunctival epithelium and GC predominantly express TGFβ2 isoform (8). The integral role of TGFβ in regulating immune response is well-documented and activation of its latent form is known to provide a crucial layer of regulation that controls TGFβ function (28). Conjunctival GC depend on TSP-1 to activate their latent TGFβ2 as, unlike other isoforms, latency-associated peptide (LAP) of TGFβ2 does not contain RGD sequence and therefore cannot be activated by integrins (8, 28, 29). In our experiment, flagellin stimulation of GC did not result in significant change in TGFβ2 message at lower concentrations tested with only increase detected at the highest concentration (**Supplementary Figure 2**). However, flagellin stimulation resulted in reduced expression of TSP-1 both *in vitro* and *in vivo* that is consistent with reduced levels of active TGFβ detected in culture supernatants of flagellin-stimulated GC. Moreover, the observed correlation of the reduced expression of TSP-1 in conjunctival epithelium with the development of chronic ocular surface inflammation in this study is consistent with our previously reported observations in human subjects (1). In individuals carrying single-nucleotide polymorphism in TSP-1-encoding gene, TSP-1 expression in conjunctival epithelial cells is reduced and correlates with their developing chronic ocular surface inflammation. Together, these findings confirm the significance of TSP-1 mediated TGFβ activation in maintaining homeostasis in ocular mucosa.

Environmental factors are considered possible contributors to the induction of autoimmunity. The relevance of microbes to chronic ocular surface inflammation associated with Sjögren’s syndrome is supported by reports of altered microbial colonization at the ocular surface in patients as well as in the mouse model of the disease (10, 11). However, it is not known how ocular surface microbes may contribute to chronic inflammation. Our study provides the first evidence of TEDs in ocular mucosa that has been described as a mechanism of microbial sampling in intestinal and respiratory mucosa (5, 30). Considering that TEDs are observed in conjunctiva with increased microbial colonization, they are likely regulated by epithelial responses to microbial stimuli as noted in intestinal mucosa (20). Our focus on GC in this study is based on their previously reported immunomodulatory function (8) and critical role in induction of immune tolerance related to the ocular mucosa (2). However, responses of stratified epithelial cells to microbes and their contribution to overall tissue homeostasis and TED formation need further investigation. The proximity of DC to GC in our study does suggest a potential role of GC responses to microbial stimulation in inducing TED formation. Furthermore, our failure to detect TEDs in normal conjunctiva tissue indicates their relevance to inflammation. Particularly, pre-existing TSP-1 deficiency in the conjunctiva with increased microbial colonization suggests that altered tissue homeostasis may drive TED formation in DC. Therefore, it is quite possible that DC in the ocular mucosa capture ocular surface antigens *via* TED formation to induce an inflammatory adaptive immune response. Their ability to migrate to draining lymph nodes and characterization of inflammatory effectors induced remain to be determined in future studies.

Our findings may appear to contradict the previously reported protective effect of topically applied flagellin on the development of microbial keratitis (31). However, there are several differences between the two studies. A major difference being that microbial keratitis represents acute inflammation, while our study addresses development of chronic ocular surface inflammation. Moreover, induction of microbial keratitis involves prior disruption of corneal barrier and much higher concentration of flagellin (500 ng *vs.* 10 ng in our study) was applied to the corneal barrier disrupted with needle injury. Thus, while the microbial keratitis study focuses on responses of damaged corneal epithelial cells typically targeted by the causative pathogen, our study highlights responses of healthy conjunctival epithelial cells.

In conclusion, we demonstrate in this report that conjunctival GC express functional TLR5 that mediates signaling leading to disrupted ocular mucosal homeostasis. The TLR5-mediated changes to the tissue environment activate local antigen-presenting cells and result in the development of chronic ocular surface inflammation. Thus, GC response to ocular surface microbes represents an important regulator in the maintenance of ocular mucosal homeostasis.

## Data Availability Statement

The raw data supporting the conclusions of this article will be made available by the authors, without undue reservation.

## Ethics Statement

The animal study was reviewed and approved by Boston University Institutional Animal Care and Use Committee.

## Author Contributions

Conceptualization, funding acquisition, resources and supervision, SM. Investigation and formal analysis, AL and LC-R. Writing—original draft preparation, AL and SM. Writing—review and editing, AL, LC-R, and SM. All authors contributed to the article and approved the submitted version.

## Funding

This research was funded in part by National Eye Institute Grant EY015472, Massachusetts Lions Eye Research Fund (MLERF) and and Sjögren's Foundation.

## Conflict of Interest

The authors declare that the research was conducted in the absence of any commercial or financial relationships that could be construed as a potential conflict of interest.

## Publisher’s Note

All claims expressed in this article are solely those of the authors and do not necessarily represent those of their affiliated organizations, or those of the publisher, the editors and the reviewers. Any product that may be evaluated in this article, or claim that may be made by its manufacturer, is not guaranteed or endorsed by the publisher.

## Supplementary Material

Supplementary Figure 1 Trans-epithelial dendrites are not detected in mice with normal or low microbial frequency at the ocular surface. Confocal microscopy images of the conjunctival explant from WT (C57BL/6) mice stained for MUC5AC (red) to label GCs and CD11c (green) to label DCs. Images show three-dimensional view of 63 z-stack images captured using laser-scanning confocal microscope. **(A)** Bottom view of the explant shows sub-epithelial location of some DCs (green), **(B)** In the top view extensions of DCs (green) and their proximity of GCs (red) is not observed. Digital magnification of the boxed region in the top panel is shown to better illustrate the absence of a DC extension.

Supplementary Figure 2 Changes in TGFb2 transcript levels after flagellin stimulation of GC cultures. The expression of TGFb2 after stimulation with indicated concentrations of flagellin was determined by real-time PCR, Data are expressed as Mean + SEM, n=4, *p < 0.05.
