# Supplementary figures and images for: Conjunctival Goblet Cell Responses to TLR5 Engagement Promote Activation of Local Antigen-Presenting Cells
Source: Front Immunol. 2021 Aug 9;12:716939. doi: 10.3389/fimmu.2021.716939 (PMC8380822; doi:10.3389/fimmu.2021.716939)

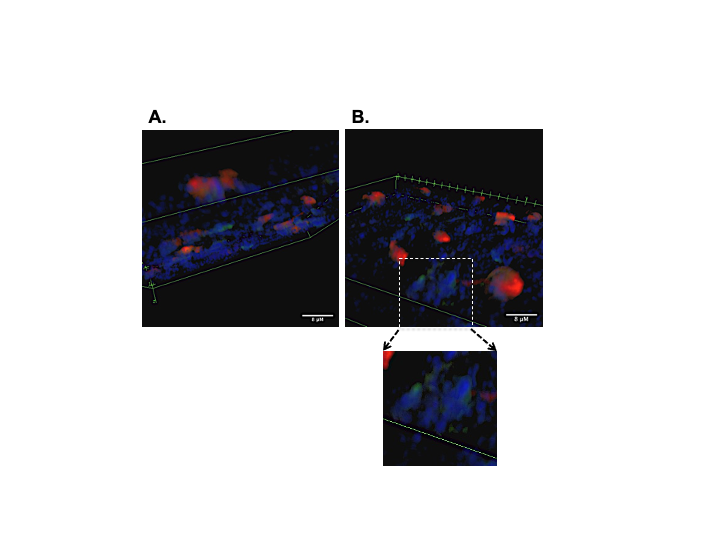

Supplement: Supplementary Figure 1 — Trans-epithelial dendrites are not detected in mice with normal or low microbial frequency at the ocular surface. Confocal microscopy images of the conjunctival explant from WT (C57BL/6) mice stained for MUC5AC (red) to label GCs and CD11c (green) to label DCs. Images show three-dimensional view of 63 z-stack images captured using laser-scanning confocal microscope. (A) Bottom view of the explant shows sub-epithelial location of some DCs (green), (B) In the top view extensions of DCs (green) and their proximity of GCs (red) is not observed. Digital magnification of the boxed region in the top panel is shown to better illustrate the absence of a DC extension. [file Image_1.tiff]

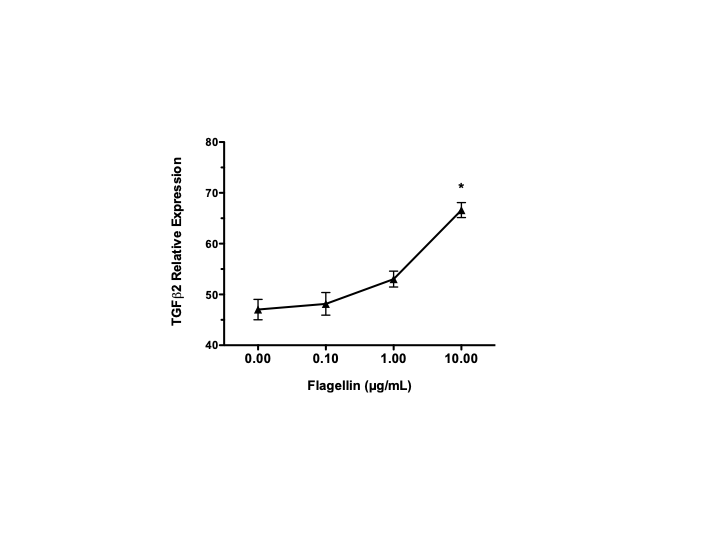

Supplement: Supplementary Figure 2 — Changes in TGFb2 transcript levels after flagellin stimulation of GC cultures. The expression of TGFb2 after stimulation with indicated concentrations of flagellin was determined by real-time PCR, Data are expressed as Mean + SEM, n=4, *p < 0.05. [file Image_2.tiff]
